# Stepping up as confident caregivers and emerging leaders of care: exploring third-year nursing students’ experiences of mentoring first-year peers in nursing homes

**DOI:** 10.1186/s12909-026-08588-y

**Published:** 2026-01-14

**Authors:** Sylvia Hansen, Elisabeth Hessevaagbakke, Katrin Lindeflaten, Stine Rokne Solberg, Andre Nicolai Fjeld Bachke, Daniela Lillekroken

**Affiliations:** Department of Nursing and Health Promotion, Oslo Metropolitan University, PB 4 St. Olavs plass, Oslo, N-0130 Norway

**Keywords:** Peer mentoring, Nursing education, Clinical practice, Nursing students, Nursing homes, Focus groups, Content analysis

## Abstract

**Background:**

Nursing students in their final year of study (third-year students) are on the verge of transitioning into professional communities. Educational approaches that enable and support their successful transitions should be applied and explored. One such approach involves third-year students mentoring their first-year peers during their initial educational and clinical encounters. Serving as a mentor may provide third-year students with a meaningful platform for developing the knowledge and skills essential for their early professional careers and practice. This study aims to explore the experiences of third-year nursing students mentoring first-year nursing students during a one-week inspirational clinical placement in nursing homes.

**Methods:**

This study employed a qualitative exploratory research design. Data collection took place in October and November 2024 using focus group interviews. A total of 60 students in their third year of the bachelor’s program at the Oslo Metropolitan University participated in nine focus group interviews. The data were analyzed using conventional content analysis.

**Results:**

The qualitative content analysis revealed one main theme: ‘Cultivating a sense of professional self: navigating the dual role of learner and mentor in clinical nursing education’, reflecting how mentoring junior peers contributed to the shaping of third-year students’ professional identity. The main theme is supported by three themes: (i) ‘From mentee to mentor – stepping into a new role’, which describes how taking on mentoring responsibilities encouraged third-year students to practice leadership, articulate their knowledge, and model quality care, fostering growing confidence and accountability; (ii) ‘Building confidence through teaching and reflection’, which illustrates how teaching skills and engaging in reflective discussions helped third-year students broaden their understanding of residential care and begin to recognize a broader view of professional nursing; and (iii) ‘Adapting to interpersonal challenges’, which highlights how managing relational difficulties encouraged teamwork, resilience, and self-awareness, supporting third-year students in developing an understanding of the relational and collaborative nature of nursing.

**Conclusion:**

This study highlights that mentoring first-year peers was a valuable learning experience for the third-year students, offering them relevant opportunities to build confidence, develop leadership skills, and strengthen their professional nursing identity. Despite the brevity of the learning activity, mentoring contributed to processes that could support their transition into professional nursing roles. These findings underscore the importance of integrating thoughtfully designed mentorship programs into nursing curricula to better prepare students for the workforce.

**Supplementary Information:**

The online version contains supplementary material available at 10.1186/s12909-026-08588-y.

## Background

Nursing education plays a pivotal role in preparing students for the complexity of nursing practice [1]. In recent years, peer student mentorship has emerged as a pedagogical approach in nursing education to increase students’ confidence, knowledge, professional growth, and preparedness for the nursing role [2]. Mentoring plays a pivotal role in nursing, as supportive and reciprocal relationships, along with the passing on of knowledge and skills, are fundamental practices that foster continuous learning cycles in the profession [3]. Peer student mentorship is therefore a relevant initiative for preparing students for professional life and practice [4].

Reviewing relevant literature of mentorship programs offered in nursing education, Rohatinsky et al. [5] proposes an understanding of the concept of peer student mentorship as a mutually beneficial formal partnership between upper-year students, in our context third-year students enrolled at the bachelor’s program (mentors), and first-year nursing students (mentees), whereby mentees are socialized into the nursing profession and are guided in technical, cognitive, academic, or organizational skills by the mentors. Peer mentorship programs can be organized in various ways, each with a unique configuration [6]. The traditional dyad model pairs one mentee with one mentor. Other configurations include one mentor supporting a group of mentees, pairs of mentors working together to mentor one or more mentees, or groups of mentors collaborating to guide one or more mentees [6, 7]. In this article, the understanding of Rohatinsky et al. [5] regarding student mentors and mentees will be applied, focusing on interactions between the two parties, whether internal or external, and whether individually or in various group constellations.

The benefits of peer-student mentorship in nursing education are often linked to learning philosophies that promote learners’ active participation within communities [8]. Learning and development in nursing occur in psychosocial, cultural, and physical environments where interpersonal relationships are strong drivers of nursing students’ learning both on and off campus [9, 10]. Peer student mentorship can particularly facilitate collaborative learning – the active, social, contextual, and engaging educational experiences gained through interactions with peers – leading to an inclusive learning atmosphere that serves as a platform for knowledge and skill transfer [4, 11, 12]. The social connection and belonging that peer student mentorship creates are effective means of reducing attrition caused by stress and anxiety in nursing students [13]. Peer mentorship programs in nursing foster a collaborative effort among students to understand, analyze, critique, and resolve professional questions. This boosts their confidence and increases their ability to navigate academic challenges and career decisions, in turn contributing to personal development and preparedness for academic success and future professional roles [14, 15] as well as to nursing communities of lifelong learners [12].

In nursing education, peer mentorship programs are primarily implemented to socialize mentees into the study program, expose them to professional norms, values, and expectations, and enhance their success across three specific domains: theoretical studies, skill laboratory training, and clinical placement [5]. This study focuses on peer student mentorship in clinical placement, more specifically, the experiences of third-year students, the mentors, who are immersed in mentoring first-year students, the mentees, by introducing them to real-world residential care in nursing homes.

Nursing education in Norway follows the European Union (EU) directives and the standards set by the Bologna Process, in which the bachelor’s and master’s degree structure is the norm [16]. A bachelor’s degree requires 180 ECTS credits, and a master’s degree requires an additional 120 ECTS. The Norwegian bachelor programs in nursing include a three-year period, with a minimum of 4,600 h of combined theoretical and clinical training, of which at least 2,300 are used in clinical placements. In line with EU requirements, theoretical and practical learning alternate throughout the three-year bachelor program, allowing students to integrate knowledge through lectures, seminars, workshops, and varied clinical experiences [16, 17]. Upon completion, students earn a bachelor’s degree in nursing and qualify as registered nurses (RNs) by demonstrating competence across three nationally defined domains: knowledge, skills, and general competence.

In the bachelor program at the Institute of Nursing and Health Promotion at Oslo Metropolitan University, third-year nursing students mentor their junior peers in several ways. In the third year of their studies, nursing students complete the clinical course ‘Nursing Patients with Complex Health Challenges’ (SYKPRA60) in nursing homes [18]. Among the intended learning outcomes is the ability to understand and apply principles of learning, mastery, change processes – and the supervision of patients, relatives, peers, and healthcare personnel employed in nursing homes. As a mandatory assignment during their clinical placement, third-year students plan and provide supervision to a group of first-year students, in collaboration with a nurse preceptor and a university nurse educator. This structure enables the use of peer mentoring as a pedagogical approach for both third- and first-year student groups during the nursing home clinical placement and involves both dyadic and group peer-mentoring elements.

Clinical placement in nursing education is crucial for students to develop the competence and confidence necessary to provide high-quality care [19]. Placement in nursing homes offers nursing students rich learning opportunities to develop their competence, given the complexity of nursing home residents’ care needs [20]. Within the Norwegian context, studies have demonstrated that nursing homes offer rich opportunities for early clinical exposure and peer-to-peer and student-to-student collaboration [21] and that the nursing home environment supports structured supervision, reflection, and meaningful student learning [22].

In Norway, nursing homes are operated by the municipalities and are typically situated within residents’ local communities [23]. Compared to several other European countries, Norway has staffing levels in these facilities that are more than twice as high, and the majority of employees hold formal qualifications [23]. Ensuring safe, reliable, and high-quality care is a key priority, with an emphasis on minimizing errors and adverse events. The Regulation of Quality of Care governs the delivery of services in nursing homes [24]. It mandates that residents’ essential physical and psychosocial needs be safeguarded and that their dignity, self-respect, and opportunities for autonomy in daily life be upheld.

While a growing body of research highlights students’ experiences in peer mentorship programs across various clinical settings, little is known about how the peer mentoring process benefits third-year nursing students who mentor first-year students in nursing homes.

A recent qualitative study examined nursing students’ experiences with peer-assisted learning (PAL) in hospital wards [25]. The study found that acting as a peer mentor strengthened upper-year students’ professional identity and commitment to nursing values. Furthermore, their clinical confidence improved, as did their ability to work in partnership and provide constructive feedback. The experience prepared upper-year students for the collaborative nature of healthcare practice and future leadership roles. The study highlights the potential for broader implementation and exploration of PAL in diverse nursing education systems and clinical settings. Similar findings are confirmed by other studies.

A literature review [5] that outlines the elements and outcomes of peer mentorship programs in nursing practice across various clinical settings demonstrates that mentoring junior peers enhances upper-year mentors’ leadership skills. These skills include providing constructive feedback, delegating tasks, coordinating and organizing care, problem-solving, and decision-making. Additional findings from the review indicate that student mentors experience increased confidence in their abilities, knowledge, self-esteem, and understanding of the nursing role. Their proficiency in fundamental nursing skills improves as they review the required material to demonstrate these skills accurately. Furthermore, the student mentors are often better prepared to seek out mentoring relationships after graduation, and their interest in pursuing advanced degrees and faculty positions is heightened.

A meta-synthesis of qualitative findings regarding student nurses’ experiences with peer mentoring programs in clinical placement across various clinical settings [26] reveals that upper-year peer mentors themselves become more active learners by discussing and demonstrating nursing skills to mentees, making their clinical studies more meaningful. Their mentoring helps them strengthen both their academic and clinical decision-making skills. The overarching theme of the meta-synthesis is that mentoring junior peers strengthens the upper-year students’ road to becoming professional nurses. However, the study demonstrates that mentors sometimes feel uncertainty about their ability to mentor, questioning whether their interactions with mentees are ‘good enough’. The findings also highlight challenges student mentors face, such as perceiving mentees’ lack of interest in highly relevant clinical situations.

Only one study included in the meta-synthesis, also included in the mentioned literature review [5], was conducted in a nursing home setting – a qualitative study that explored nursing students’ experiences with peer mentorship [27]. The study procedure involved pairing first-year students with upper-year students on their first day of providing Activities of Daily Living (ADL) care to nursing home residents. During the mentoring activity, student mentors performed tasks such as transferring residents from bed to wheelchair, helping them with oral care, bathing, dressing, feeding, and taking vital signs, while mentees observed. Gradually, the student mentors encouraged mentees to assume greater responsibility, guiding them in the principles of care provision. The peer mentors in this study expressed that the opportunity to assume a leadership role and explore new roles in the clinical setting was a welcome change for them. They had not previously been granted the role of ‘the person who knows more’ in their clinical placement portfolio [27].

Despite the recognized advantages of peer mentorship in clinical nursing education, relatively little research has explored the experiences of third-year nursing students in their mentoring roles, particularly in the context of clinical placements in nursing homes. As third-year students enter their final year of study, they are at a pivotal stage as their transition into the nursing workforce is near forthcoming; therefore, a better understanding of their educational needs, which enables successful transitions, is essential. This study explores the experiences of third-year nursing students enrolled in a course that requires peer mentoring as a mandatory assignment, with third-year students serving as mentors to first-year students during a one-week inspirational practice in nursing homes. By doing so, the study aims to advance knowledge on assessing the integration of such a peer mentorship program into clinical nursing education.

## Methods

### Design

This study employed a qualitative, exploratory design guided by Creswell [28] and used content analysis to analyze empirical data from focus group interviews with third-year students. The design provided researchers with the flexibility to discover new insights and understand the experiences of third-year students mentoring first-year students, experiences that are relatively underexplored in prior nursing home research as a learning environment. The Consolidated Criteria for Reporting Qualitative Research (COREQ) checklist was used to ensure transparent reporting of the qualitative research [29] (Supplementary file 1).

### Aim of the study

This study aimed to explore the experiences of third-year nursing students serving as mentors to first-year nursing students during a one-week inspirational practice in nursing homes.

### Sample, recruitment, and context

The participants in this study were nursing students enrolled in their third and final year of the bachelor program in the Department of Nursing and Health Promotion at Oslo Metropolitan University [18]. Recruitment was initiated by the course coordinators (two of the researchers and coauthors of the manuscript), who informed all third-year students (*N* = 331) about the study both verbally and by email and invited them to participate in one focus group interview. The invitation was followed by several reminder emails sent by nurse educators and researchers involved in the project. However, of the 331 third-year students, only 60 participated in the study. The sample comprised 55 women and 5 men, aged 20 to 31. Of these participants, 24 had prior work experience in the healthcare field. The focus groups varied in size: one group included five participants, four groups had six participants each, two groups had seven participants each, one group had eight participants, and one group had nine participants. Only five focus groups had one male participant each.

During the interviews, the students were engaged in clinical placement in the course ‘Nursing Patients with Complex Health Challenges’ (SYKPRA60) [18] in nursing homes. During their clinical placement, they planned and conducted mentoring of a group of first-year students attending a one-week inspirational practice. Due to the large number of students enrolled in the bachelor program, the cohort was divided into two groups for clinical placement to ensure sufficient placement opportunities in nursing homes. As a result, the clinical period was conducted in two consecutive rounds. The first group comprised 168 third-year students who mentored 233 first-year students during the fifth week of their placement, while the second group comprised 163 third-year students who mentored 230 first-year students during the third week of their placement. In larger nursing homes, where more third-year students were placed, the mentoring ratio typically ranged from 2 first-year students to 1 third-year student. In smaller nursing homes, however, mentoring could also occur on a 1:1 basis.

To be eligible for participation, students had to meet the following criteria: (i) be enrolled in the 2024–2025 academic year, (ii) participate voluntarily, and (iii) agree to being recorded during the interviews. Those interested in participating were informed to contact the researchers via email to schedule an interview. For practical reasons, the researchers considered forming focus groups of students from the same nursing home to ensure participants felt comfortable and at ease, thereby creating a familiar, supportive environment during the interviews, as they knew one another.

### Data collection

The data were collected over the two clinical periods. The focus group interviews took place between October and November 2024, allowing students from both groups to participate. A total of nine focus group interviews were conducted, with four groups comprising third-year students from the first cohort and five groups consisting of third-year students from the second cohort.

Focus group interviews, as a data collection method in this study, were chosen for their efficiency because they enable the exploration of diverse perspectives, with participants building on one another’s statements, thereby generating new insights and information [30].

The focus group interviews were guided by a semi-structured interview guide developed by the research team prior to data collection. The interview guide was informed by existing literature that highlights the importance of peer mentoring in clinical nursing education, the development of observational and ethical competencies in nursing homes, and students’ transition from learners to mentors. These insights helped shape the structure and content of the questions to ensure they were relevant to the study’s aims and captured the complexity of students’ experiences. Table 1 provides the semi-structured interview guide used during interviews.

**Table 1:** Semi-structured interview guide for participants’ experiences

No	Interview questions
1.	Please describe how you prepared for peer mentoring first-year students during the inspiration practice placement. What did you emphasize? Do you have any suggestions for improvement?
2.	Please describe how you prepared the welcome and introduction for first-year students on the first day of the inspiration practice placement.
3.	How would you describe peer mentoring as a learning activity? What areas did you emphasize during mentoring? Can you share some examples?
4.	Two years ago, you received mentoring from third-year students during the inspiration practice. Can you describe what it was like to be the mentors for first-year students this time?
5.	Please describe what you believe are the benefits of being mentored by third-year students. What challenges might this involve?
6.	Please describe your experiences with the implementation of the mentoring of first-year students in the inspiration practice.
7.	Please describe what you enjoyed most and least about mentoring.
8.	Can you describe what you learned most from mentoring first-year students?
9.	Please describe your experiences with the supervision you gained from nurse educators and nurse preceptors.
10.	Please describe how mentoring first-year students relates to your current studies and future career as a registered nurse.

Although each interview was scheduled to last up to 60 min, actual durations ranged from 42 to 53 min. All the interviews were conducted at the end of the workday in a quiet room at the nursing homes, where students were completing their clinical period. Each interview was conducted by two nurse educators, in pairs, all of whom are authors of the present article, with one serving as the moderator and the other as the co-moderator. The nurse educators who moderated focus group interviews, though employed in different units within the department, were familiar with one another. Some of them also recognized students from previous years of the program. Additionally, some third-year students in this study had previously participated in a related study as first-year students. The previous study aimed to explore first-year nursing students’ experiences of being mentored by third-year students during a one-week inspiration practice in nursing homes [31] and their perceptions of the nursing profession in that context [32].

The moderator was responsible for posing questions and following up on students’ descriptions, while the co-moderators’ role involved taking notes, observing group dynamics, and managing the recording device. During the interviews, participants were encouraged to use everyday language, share their thoughts and subjective experiences as mentors for first-year students, and offer suggestions for course improvements. As a result, participants offered in-depth, meaningful responses, resulting in detailed, nuanced expressions of their perspectives.

The interviews were recorded digitally and transcribed verbatim using a smartphone app (Diktafon), with audio files sent to “Nettskjema,” a university-approved application, to ensure secure storage and data protection. This app securely stores encrypted interviews on a server to safeguard privacy and data. Although the interviews were automatically transcribed by the app, some minor revisions were made to certain words and sentences to improve clarity and accuracy.

### Data analysis

The data were analyzed using conventional content analysis, drawing on the approach described by Hsieh and Shannon [33] and guided by the analytical steps outlined by Graneheim et al. [34] and Graneheim and Lundman [35]. This approach is well-suited to exploring participants’ experiences and identifying latent patterns in textual data through systematic coding. All authors participated in data collection, thereby facilitating deep familiarity with the material prior to analysis.

Although all interviews were digitally recorded and transcribed verbatim using the Diktafon smartphone app, the authors carefully checked each transcript for accuracy by repeatedly listening to the recordings while reading the text, correcting minor errors, and ensuring that the transcripts provided a trustworthy foundation for analysis.

The analytical process began with each author independently reading the transcripts several times to develop a comprehensive understanding. Meaning units relevant to the study aim were then identified, condensed, and assigned initial codes. No codes were predetermined; all were generated inductively from the data. The research team subsequently met to compare and discuss their coding, resolve discrepancies, and reach consensus on shared interpretations. Through an iterative process of reflection and comparison, codes with conceptual similarities were grouped into categories and subcategories, which were then abstracted into broader themes. Continued movement back and forth between the transcripts, meaning units, and emerging codes ensured that the developing themes remained grounded in the data.

To enhance analytical rigor, the team engaged in ongoing reflexive discussions throughout the analytic process and documented analytic decisions to create an audit trail. Researcher triangulation, where multiple authors contributed to coding, categorization, and theme refinement, further strengthened credibility and dependability. Although researcher triangulation was applied, the first and last authors were primarily responsible for conducting the analysis. The final analysis yielded three sub-themes and one overarching theme that captured the core interpretation of participants’ experiences. Table 2 provides an example of meaning units and their corresponding codes in the development of the first sub-theme.

**Table 2:** Example of coding tree for the first sub-theme “From mentee to mentor – stepping into a new role”

Meaning units	Code	Sub-theme
“Being in the situation, and demonstrating, it was like … we must make sure to do it the proper way so that they [mentees] learn it right” (Participant, FG4) “We had to justify our care actions and be independent, instead of just following our preceptor” (Participant, FG6) “We wanted to display what makes up a pleasant and dignified groom for the patients” (Participant, FG6)	Taking on new responsibilities	From mentee to mentor – stepping into a new role
“They [mentees] might have questions that we had to answer. So, it was important to prepare well” (Participant, FG3) “We focused on sharing ideas within the mentor group, preparing to manage in a good manner” (Participant, FG6)	Preparing to fill a new role	
“You are overly cautious that they [mentees] are observing what you do, so you become very aware of how you do things” (Participant, FG9) “You become reminded of the small things that mean a lot, how things should be done” (Participant, FG6) “You must perform; they [mentees] absorb everything they see” (Participant, FG6)	Becoming role-models	
“Trying it out, you get to know yourself better … how will I be as a mentor and a future nurse?” (Participant, FG8) “It’s nice to feel more independent; it makes you realize you are moving forward” (Participant, FG9)	New experiences shaping nursing identity	

Participant quotations, along with corresponding code numbers for the focus group interviews, are included to support the findings.

### Ethical perspectives

Before starting data collection, approval from the Norwegian Agency for Shared Services in Education and Research (SIKT, Ref. nr. 334855) and the leader of the Department of Nursing and Health Promotion at Oslo Metropolitan University was secured. The study was conducted in accordance with the principles of good ethical practice in scientific research, as described in the Declaration of Helsinki [36], including informed consent, confidentiality, and consideration of potential consequences.

All participating students provided written informed consent before the interviews commenced. The students were informed about the study’s aim and data collection method and assured of confidentiality. They were informed that their participation in the study would not result in any financial or other benefits (e.g., improved exam grades). The students were also informed that they would have one week to decide whether they wished to participate. Additionally, they were given the option to withdraw from the study at any time without consequences for their clinical placement assessment or further education at the university.

Two of the researchers and co-authors of the manuscript, who also selected and moderated the focus groups, were coordinators of the clinical course and supervised students’ placements in nursing homes, meaning that some participants were already familiar with some of the moderators prior to data collection. Given that nurse educators may be viewed as authority figures, students might have felt vulnerable and answered ‘Yes’ when invited to participate in focus groups. Therefore, to avoid undue influence as moderators, the course coordinators served as co-moderators during the focus group interviews, while the other researchers served as the moderator. The research team, employed as nurse educators at the university, strived to be as transparent and open as possible with participants, reminding them prior to each focus group that participation in the study was voluntary and that their answers should not convey ideas that would fit the interviewers’ expectations. Participation in the study was clearly communicated as voluntary, and students were repeatedly assured that their decision to participate or to withdraw at any time would have no consequences for their grades, evaluations, or progression in the course. The distinction between the educators’ teaching roles and their role as researchers was emphasized, and students were informed that their responses would be anonymized and not shared with course faculty in a way that could be linked to them personally. These measures were intended to reduce the potential impact of the inherent power imbalance and support an environment in which students felt safe to share their genuine perspectives. Furthermore, the students were assured that the interviews would be conducted as a dialogue about their subjective experience with mentoring first-year students, and not as an assessment of the knowledge they gained during clinical placement. No participants who initially consented to take part subsequently withdrew from the study. All the participants provided written informed consent before the focus groups.

The interviews were recorded using the Diktafon app, which presents certain limitations, including potential vulnerabilities related to data security and reliance on stable device performance. To safeguard participants’ privacy, all recordings were stored on Nettskjema, an encrypted, university-approved application, and transferred immediately after each interview to a password-protected institutional computer. The interviews are kept safely in accordance with Oslo Metropolitan University’s guidelines and regulations for data research storage. Participants’ names are omitted from the transcripts to ensure anonymity. The transcripts are stored in a locked cabinet and are accessible only to the researchers involved in the present study. Once the research project is completed and the results are published, all audio files and interview transcripts will be permanently deleted.

### Rigour of the study

To ensure the rigor of the study, the researchers followed established guidelines for conducting focus group interviews [30] and analyzing qualitative data [33]. Six researchers were involved in the research process, each facilitating two or three focus groups in pairs. Working in pairs increased consistency, as the same interview guide was used across all groups, and the facilitators met regularly to discuss the process and ensure a uniform approach. Conducting the focus groups with multiple facilitators also enabled the research team to incorporate diverse professional perspectives into the analytical process, thereby reducing individual bias and strengthening the credibility of the findings [37].

Rigor was further enhanced through prolonged engagement with the data. All transcripts were read repeatedly, and the researchers first developed codes and categories independently and then collaboratively through consensus-based discussions. This ensured that the interpretations were firmly grounded in the data and reflected the participants’ experiences [38]. Throughout the process, the research team maintained a close relationship with the data, revisiting transcripts and initial interpretations to avoid overlooking essential themes relevant to the research question.

Trustworthiness was supported by transparency in the analytical steps and by providing detailed descriptions of the context, sample size, and research process. These descriptions enable readers to evaluate the transferability of the findings to other settings or populations. However, in qualitative research, transferability is not determined by sample size but by the extent to which readers can judge the applicability of the findings to other contexts. To facilitate this, we provide detailed descriptions of the study setting, participants, data collection method, and analytical steps, enabling readers to assess whether the findings may be relevant to similar educational environments. This approach aligns with Lincoln and Guba’s [39] criteria for trustworthiness, which emphasize thick description as the foundation for transferability [39]. Finally, the clear documentation of the study’s methodological decisions and analytic procedures strengthens the study’s reliability and increases the likelihood that similar findings would emerge in similar contexts [40].

## Findings

The qualitative content analysis revealed one main theme: ‘Cultivating a sense of professional self: navigating the dual role of learner and mentor in clinical nursing education’. This theme illustrates how the third-year nursing students navigated and balanced their learning and the mentoring of their first-year peers. Through the rewards and challenges of mentorship, the third-year nursing students developed more confidence as care providers and gained emerging leadership skills, shaping their professional identity and deepening their understanding of nursing within the context of residential care. The main theme is supported by three sub-themes: (i) ‘From mentee to mentor – stepping into a new role’, (ii) ‘Building confidence through teaching and reflection’, and (iii) ‘Adapting to interpersonal challenges’. These three sub-themes reflect the transformative processes that instilled a deeper sense of professional identity in the third-year students through peer mentorship.

### From mentee to mentor - stepping into a new role

This sub-theme highlights the transition of the third-year students from being mentees in clinical placement themselves to taking on a mentoring role, mentoring their first-year peers. Mentoring gave the third-year students new responsibilities and new experiences which encouraged them to practice and demonstrate high-quality care, and challenged them in ways they were not accustomed to in their previous clinical placements. One of the participants said:

… is a bit unfamiliar to me, so to speak, because I have only been here for a couple of weeks. But trying to combine these two roles – both as a student in placement and also as a student in a mentoring role – and making it credible for them [mentees] … is challenging but exciting … (Participant, FG3).

Most of the participants associated their new role as mentors with the one of a preceptor, like they had gotten to know the role of a preceptor in their prior clinical placement and strived to mimic that role. Just as they had followed and learned from their preceptors, their mentees now followed and learned from them. One of the participants said:

It’s not like you are the boss [when mentoring]. But now that we were the ones responsible, instead of us following our preceptors, they [mentees] followed us. So, it was a new experience and different for us, and challenged us to justify everything we did just like a preceptor should (Participant, FG6).

However, the participants also saw themselves as different from preceptors since their prerequisites were not the same. As they said, they were students themselves and not trained to mentor others, the mentees had different expectations of them compared to fully qualified nurse preceptors, and they were not very acquainted on their wards. Before meeting the mentees, some participants had already been in clinical placement for four weeks, while others had been students in the nursing home for only two weeks. Some participants found it challenging to serve as mentors when they were still unfamiliar with staff and routines and did not yet know the residents’ needs and circumstances. However, most participants said that they managed to combine their relative unfamiliarity with the nursing home setting with mentoring mentees in hands-on resident care, drawing on their prior experiences, being pragmatic, and communicating effectively. One of the participants said the following concerning taking on mentees early in the placement period:

It felt a bit unnatural showing them [mentees] around the nursing home when I myself could have benefited from or needed to be shown around (Participant, FG5).

Responding to this point of view, another participant said:

It’s not that I disagree with you … At the same time, at least in my experience, we learned a great deal from it. And they [mentees] didn’t have very high expectations of us in that regard either, since they knew well that we had not been in placement for long – we explained it to them on the first day, and that it’s not like we know everything. They understood that we didn’t always know where to find the equipment needed and didn’t know residents well (Participant, FG5).

Some of the respondents mentioned that in their opinion it would have been preferable that the mentor activities were scheduled later in their placement period, as they believed they would be better acquainted with residents then and could have offered even more guidance to mentees.

The independence that came with the mentor role was new and exciting to the participants. Throughout the focus groups, participants consistently discussed the benefits of their independence in the mentoring role and expressed positive reinforcement and pride being granted it. One respondent said:

It was fun to do something independently. And I didn’t feel that we needed to be supervised by teachers or by staff … We had continuous dialogue together in the mentor group, supervising each other … We used a lot of ‘well done’ and ‘thumbs up’ and sometimes ‘You did that well, but next time, try … and use more time’ (Participant, FG3).

To manage their new role, and to earn their independence, the participants expressed that they prepared themselves within their mentor teams to provide mentees with meaningful learning activities. Their preparations, having a tangible goal, made their effort seem purposeful. Also, some of their preparations, like repeating curriculum and clarifying the scope of mentees learning enabled them to learn more and engage more intentionally with the complexities of caring for older adults. Indeed, the participants highlighted that they sought to demonstrate high-quality nursing, which both encouraged them to prepare academically and to be more mindful of appropriate language and professional and ethical conduct, thereby presenting themselves as role models. One of the participants said the following about demonstrating quality care to mentees:

We wanted to show them what is essential. When we showed them the care procedures, we took it step by step, emphasizing that we were also creating a sense of security for the residents, not just performing a task (Participant, FG5).

Another participant emphasized that mentoring made her more conscious of her conduct:

It [mentoring] means you must be quite pedagogical when speaking. I had a lot of thoughts about how I presented myself before residents and spoke to them; it was important that it was right (Participant, FG9).

The transition from mentee to mentor marked a shift in the participants’ professional identity, as they felt obliged to ‘deliver’. One of the participants said:

I like the good feelings that come afterwards when you receive feedback, like, ‘Oh, you did that well; you spoke well in that situation’. And you feel more confident in your role … I mean, when you suddenly become the one who is supposed to show and explain, then you must deliver … (Participant, FG6).

Their new responsibilities challenged the participants to articulate their knowledge and demonstrate skills with confidence. Some participants felt the experience facilitated their leadership abilities. One of the participants said:

This is, in a way, one of the few opportunities in our clinical portfolio where we actually get to be hands-on and feel leadership. So, it was very useful (Participant, FG4).

The experience of managing their mentor role reinforced the participants’ learning, instilling greater accountability and self-assurance, as some of them noted, and was associated with a significant shift in their perceptions of their future roles as fully qualified and registered nurses. One of the participants said:

It has given me many reflections on how I want to be as a nurse. I believe it is essential to be inclusive and collaborative to create a safe environment. Now that I have seen that I can fill that role, I am actually looking very much forward to it (Participant, FG1).

### Building confidence through teaching and reflection

This sub-theme focuses on how mentoring provided the third-year students with opportunities to apply and reinforce their nursing knowledge and skills. It underscores how explaining concepts, demonstrating tasks, and reflecting on experiences and care options contributed to the refinement of the third-year students’ understanding of nursing practice and to increase their self-confidence. In turn, this strengthened the development of their professional identity.

Engaging in preparation, providing demonstrations of care, and supporting mentees in their care delivery allowed the participants to apply their knowledge in practical real-life situations. Many participants felt that this hands-on experience helped them sharpen their skills and advance their critical thinking. When teaching mentees fundamental nursing skills, including personal hygiene, toileting, nutrition, measuring vital signs, documentation, and effective communication with individuals living with dementia, the third-year students became more self-conscious about their professional practice and how they performed. They found themselves reflecting on how they executed these tasks, as they had to explain and demonstrate them to mentees. This process strengthened their understanding and proficiency. Two excerpts from the focus groups illustrate participants’ self-conscious reflections as they assisted the mentees:

You had to stop at each stage and ponder … Is this a justifiable way to do it? (Participant, FG4)

When you must speak rather than just acting, something happens. It is like a reminder, and there is a two-sidedness to it, really, a tightening up for me and a learning session for the mentee (Participant, FG5).

Most participants expressed a sense of coping in their roles as peer mentors. They realized they possessed more professional knowledge and skills than they initially thought, which they could pass on to their mentees. For instance, one participant reflected on the reassurance gained from already knowing fundamental nursing care before stepping into the mentor role:

It’s reassuring to have learned it [the provision of fundamental nursing care] beforehand, before being thrown into this role. So, the fundamentals of nursing skills are something you must have, I think, and it’s great that you’ve learned to provide it in a nursing home setting before (Participant, FG5).

As the participants became more aware of their own knowledge and skills, it contributed to building up their confidence. As most of them noted, teaching and reflecting on situations in the nursing home not only supported the mentees’ learning but also strengthened their own confidence and competencies. One participant said:

There are a lot of things you do not really think about being able to do, and then you realize, ‘Oh, yes, I have actually learned this over the past two years’. It wasn’t something I could do before I started nursing education. So, you might not give yourself enough credit for what you have learned and can do … (Participant, FG9).

Beyond the opportunity to become more confident in their ability to apply and rely on their knowledge and skills, the mentoring experience also offered the participants with ample opportunities to reflect on their practice concerning resident care and of the implications of their actions. Due to improvements in nursing skills and confidence that these reflections brought about, some of them felt that the duration of one week for the mentees’ placement was too brief, as they noticed their own advances mostly towards the end of the week. They believed that extending the period to two weeks would have increased their learning outcome even more.

Most of the participants spoke of how they reflected collaboratively within their mentor groups when preparing to mentor, drawing on both positive and negative experiences being mentored by preceptors or mentors themselves in prior placement periods. It was essential for them to bring these experiences with them as a compass to navigate their supervision of mentees, as their own experiences of being mentees was an important source of insight worth learning from and carrying forward. One of the participants said:

We discussed our experiences from prior placement – for good and bad. I was thinking about all the different preceptors I’ve met, what they did that I could learn from, and what I didn’t learn anything from. It’s like different worlds I’ve been in … When mentoring, we tried to focus on our positive experiences, like being given good explanations and being met with forbearance (Participant, FG2).

Indeed, the mentor group stood out as the participants’ most important support system carrying out the mentor activities. Collaboration and reflections within the group not only helped them feel more confident but also allowed them to refine and broaden their perspectives. One participant said:

Collaborating with the mentor group, we got to know each other quite well, and eventually, we started feeling comfortable speaking up and joking around. And beyond that, both during the preparation phase, and during mentoring, we were able to learn from each other and exchange ideas. We eventually felt at ease saying: ‘I think you are mixing things up a bit here’ – giving each other different viewpoints. At least on my part, it helped me discover new perspectives (Participant, FG3).

Through their group reflections, the participants also became more attuned to the caring aspects of nursing. For instance, some of them noted that some of the mentees were primarily interested in learning and performing technical procedures and expressed disappointment when they were ‘only’ spending time with residents. Within the mentor group, the third-year students discussed the importance of helping mentees reconsider what constitutes a nursing procedure. They emphasized that caring for and interacting with residents is just as integral to nursing as technical tasks. One of the participants said:

When you are completely inexperienced, you don’t fully understand what nursing is. You might think it’s about taking blood samples and similar procedures, which is highly specific. They [mentees] have not yet fully grasped that simply talking with a patient is a procedure in itself. We wanted to show them that they can make a difference for the patients (Participant, FG3).

### Adapting to interpersonal challenges

This sub-theme captures the relational aspects of the third-year students’ experiences. It explores their experiences of building supportive relationships and developing teamwork skills to navigate and become more resilient in the face of challenges that arose during the mentorship process. The sub-theme highlights that third-year students not only provided support to their mentees but also recognized the importance of leaning on and supporting one another to adapt and persevere in the face of obstacles.

Participants’ prior experience with inspirational practice helped them refine their leadership skills and strengthen their ability to tailor their guidance to the diverse learning needs of mentees. All participants expressed a strong desire to ensure the mentees felt welcomed and cared for. One participant said:

In a way, we have been in their shoes as new students on the ward in a nursing home, so we know what it’s like to arrive at a completely new place, especially since many of us had no prior experience in healthcare before starting our nursing education (Participant, FG4).

During the week, the participants sought to create a safe and supportive environment in which mentees felt comfortable asking questions, making mistakes, and learning from real-life scenarios without fear of judgment. However, they experienced difficulties and at times uncomfortable situations, which tested their resilience within the mentor-mentee relationship.

For some third-year students in the second group, mentoring was challenging since they were responsible for mentoring a large group of first-year students while still becoming familiar with the nursing home environment. In some cases, this limited their ability to build positive relationships with the mentees and left them feeling unprepared. One of the participants said:

When there is a large group of first-year students, and we [the mentor group] are also quite a few, then it can be difficult both to interact and to agree … and a bit unpredictable (Participant, FG6).

While many mentees were perceived by their mentors as engaged and motivated to learn, some were perceived as the opposite. In some focus groups, participants reported that mentoring could involve navigating difficult situations. Some mentees appeared disengaged, seemingly attending only to fulfill attendance requirements. They described instances where mentees would sit on their phones, arrive late, or even fall asleep during mentoring sessions. In some cases, mentees were not appropriately dressed for clinical work. These experiences led some third-year students to question whether they should have been expected to address such behavior without clearer guidelines or additional support. One participant said:

That responsibility shouldn’t have been ours, or we should have had clear guidelines: what do we do? It’s challenging to say ‘You can’t do this or that’ when they are our age. We are students, unlike teachers whose job and expertise are to handle such problems (Participant, FG3).

The participants said that disengaged mentees challenged their ability to persevere and highlighted instances in which they struggled to adapt. While some of them reported resolving certain situations through discussions with each other and with mentees, others experienced frustration when their efforts to intervene were ineffective or ignored.

Another challenge for some participants was the age and experience gap between mentors and mentees. Some mentees were significantly older or had more experience in nursing home settings than the mentors themselves. This made it more difficult for some mentors to build genuine connections and to feel confident in their mentoring roles. They also struggled to identify learning activities suitable for both experienced mentees and mentees with no prior work experience in the healthcare sector. One participant shared:

I found it a bit challenging to mentor, for example, two first-year students - one older than me and with a lot of experience, and one with none. And then, you somehow have to teach something new to both at the same time … (Participant, FG8).

Experiencing challenges, some participants described how they relied on and supported one another in coping with these challenges. Collaborative problem-solving fostered a sense of teamwork and connection and provided opportunities to recognize both strengths and limitations within the mentor group. This collaboration not only could mitigate some of the obstacles they faced but also contributed to their professional identity formation by exposing them to key aspects of leadership, collaboration, and communication. One participant noted:

We learned a great deal through our collaboration in both planning, implementing, and managing the mentoring tasks, even when it was difficult. We had to discuss and find solutions that everyone could embrace. We learned to have open communication and to agree to disagree (Participant, FG2).

Another participant highlighted the importance of teamwork and the dynamics within the mentor group:

I believe we collaborated very effectively. If we hadn’t, I think they [mentees] would have noticed that our dynamics were off, which could have led them to the wrong impression of the nursing role and our expectations. Therefore, it is essential that, as mentors, we are given sufficient time to become familiar with each other. It’s not guaranteed that we will feel comfortable enough to expect things from one another, give feedback, delegate responsibilities, and support one another (Participant, FG3).

While the third-year students did not always succeed in overcoming challenges, the process of trying to adapt through teamwork, peer support, and reflection on their efforts helped them begin to gain insights into their incremental capacity to manage adversity.

## Discussion

The aim of the study was to explore the experiences of third-year nursing students mentoring first-year nursing students during a one-week inspirational clinical placement in nursing homes. Third-year nursing students are at a pivotal stage as they prepare to transition into the professional nursing community. By exploring their mentor experiences during a one-week inspirational practice in nursing homes, we found that taking on the dual role of learner and mentor in clinical nursing education cultivated a sense of professional identity among the third-year students. This development rose from the process of embracing and adapting to a new role within the clinical setting, which provided opportunities to enhance their confidence as capable caregivers and emerging leaders in the care of nursing home residents.

The following discussion will be divided into two sections. The first will discuss our interpretations and reflections on how the mentoring experience contributed to third-year students’ confidence in providing nursing care. The second will discuss our interpretations and reflections on how the mentoring experience contributed to the shaping of the emerging nursing leadership abilities in them. Our discussion will also dwell on the design of the mentorship program the students took part in and its’ impact and relevance.

### Building confidence and growing as capable caregivers

The findings indicate that mentoring first-year peers in nursing homes provides third-year nursing students with valuable opportunities to build confidence and develop competence as caregivers. Confidence increased through coping with and thriving in the mentoring role, which compelled the third-year students to reflect on their knowledge, apply their skills in practice, and embrace professional accountability to provide mentees with relevant learning opportunities.

Acting as mentors encouraged third-year students to model professional behaviors and communicate effectively with residents and other stakeholders. Coping reinforced their self-assurance and allowed them to envision how they might aspire to perform as future nurses. Consistent with our findings, other studies support the idea that peer mentorship roles enhance student mentors’ confidence. Rohatinsky et al. [5] found that assuming the mentoring role encourages students to refine their knowledge, thereby enhancing their confidence as caregivers. The mentor experience makes upper-year students’ learning more intentional and focused, thereby helping them transition to a professional caregiver mindset. This phenomenon was also identified by Jacobsen et al. [26] and interpreted as a means of strengthening the pathway of upper-year students to become professional nurses.

Giordana and Wedin [27] found that mentoring junior peers in nursing homes increased upper-year students’ autonomy in caregiving and enhanced their confidence by encouraging them to integrate their knowledge and make clinical decisions independently. Echoing the findings of their study, the participants in the current study appreciated the independence that came with the mentoring role, felt proud to be entrusted with independently supervising first-year peers, and immersed themselves in demonstrating and passing on what they perceived as high-quality care to nursing home residents. This prompted their self-reflection.

Being entrusted to perform independently in the nursing role has been found to foster confidence among nursing students in other studies. A qualitative study exploring experiences that support nursing students’ development of professional identity during their final year [41] found that the opportunity to work independently in clinical settings, without a nurse preceptor present at all times, substantially increased students’ confidence. Being granted independence increased students’ reflections and enabled them to connect the dots when making clinical decisions; consequently, their confidence grew. An integrative review aiming to synthesize factors influencing the professional identity development of nursing students [42] similarly argues that being granted roles and responsibilities challenges nursing students’ thinking and appeals to their capacity to reflect on optimal care actions. Confidence, as outlined in these studies, is not a direct outcome of independence but is shaped through reflection and self-discovery that autonomous practice facilitates.

A concept analysis clarifying the meaning of the concept ‘confidence’ portrays it both as an outcome of antecedents and as a dynamic process [43]. Levels of confidence in a person can increase or decrease depending on moderating factors such as learning a new skill, experiencing fulfilment, achieving successful performances, receiving praise and encouragement. These moderating factors align with the experiences of the participants in the current study, the participants describing positive experiences like reinforcing their knowledge and skills, fulfilling the mandatory assignment successfully, and receiving praise from the mentees and each other. However, confidence is not solely a byproduct of antecedents. Confidence is fostered by experience, self-reflection, and intentional action, thereby building a robust sense of self [43]. Therefore, beyond external reinforcement, the confidence gained by the participants in our study may also be related to developmental processes facilitated by the mentoring role. The mentoring role of the participants in our study compelled them to assess their own care practices and refine their understanding of what constitutes quality nursing care. They largely served as their own judges of knowledge and skills essential to residential care and discussed and modified their subjective perceptions of care practices to be able to ‘deliver’ when mentoring.

A qualitative study exploring the meaning of confidence as perceived by nursing students [44] found that confidence relates to self-discovery, including recognizing one’s strengths and weaknesses in patient care. This also aligns with the current findings: the participants both realized they already had the relevant knowledge and skills to be proficient caregivers and mentors and acknowledged areas for improvement.

In the clinical portfolio of nursing students, few other learning activities provide comparable independence and opportunities for self-discovery. The participants’ reflections in this study strongly affirm the value of the mentorship program they undertook. To further elaborate on the mentorship program as a learning activity, the mentoring process of the participants involved both dyadic and group peer mentoring elements. Mentoring sometimes involved several third-year students jointly supervising several first-year students, depending on the situation. This structure provided participants with multiple opportunities to reflect on care practices and approaches together, thereby creating a supportive learning environment in which they could balance self-doubt with growing self-assurance.

A literature review on group mentoring [7] identifies the pooling of mentors’ knowledge and experience as a characteristic of group mentoring. The input of diverse perspectives on personal and professional learning broadens co-mentors’ perspectives and enables substantial development and growth. Since the goal of mentoring is the passing on of wisdom and experience to others, the review calls into question whether group mentoring, like many-to-many mentoring (MTMM), appears to be underutilized or underreported, considering its potential. Seemingly, group support set the stage for the participants in this study to collaborate – the mentor group serving as a vessel for the participants’ development related to clinical skills, communication, and caring practices, allowing them to increase their confidence in the care of nursing home residents. Even participants in the second placement cohort who felt the inspirational practice occurred too early in their placement period – before they had gotten acquainted with the ward, employees, routines, and residents’ needs – largely coped due to the support within the mentor group.

The MTMM perspective has not been well researched in the clinical context of nursing. MTMM, however, addresses peer-assisted learning (PAL). Results from a qualitative systematic review that synthesized the best available evidence on experiences of PAL among nursing students in clinical practice [45] indicate that students who collaborate with peers in clinical practice are perceived as providing and receiving reciprocal support and feedback. Furthermore, it enables them to develop confidence and to recognize that peer support mitigates the challenges they face. Hence, the systematic review calls for clinical practice areas that nurture both formal and informal collaboration between nursing students.

### Being empowered as emerging leaders of care

Acting as mentors to first-year peers positioned third-year students in roles in which they modelled professional skills and behaviors, provided guidance, and demonstrated what they perceived as high-quality nursing care within the nursing home setting. Through these experiences, the third-year students gained opportunities to develop their leadership skills and establish their identities as emerging leaders in the care provided to nursing home residents. These findings align with other studies reporting that upper-year students in mentorship roles in clinical contexts refine leadership abilities by giving constructive feedback, delegating tasks, coordinating care, solving problems, and making care decisions [5, 26, 27]. These activities strengthen mentors’ professional growth, a finding that echoes in the present study’s findings.

In the focus group discussions, the participants reflected on their roles as mentors, noting the pressure to ‘deliver’ as role models. This prompted them to think critically about care decisions and consider the impact of their actions. Some participants noted that this mentorship program stood out as a unique opportunity to learn leadership, as their earlier clinical placements had provided limited opportunities to develop such skills. However, based on participants’ discussions in the focus group interviews, it remains unclear how they conceptualized ‘leadership’ or what learning opportunities in leadership their prior clinical placement periods offered. It has been suggested that nursing students often hold narrow or traditional views of leadership [46]. For example, a qualitative study exploring the perceptions of leadership among third-year undergraduate nursing students before they qualified as registered nurses [46] found that these students frequently associate leadership with solo, charismatic, or authoritative figures who are detached from the realities of everyday nursing care practice, administrating or managing wards. The participant in the study [46] could not elaborate on what distributed leadership or clinical leadership might mean in practice. Francis-Shama [46] argues that inconsistencies in the manner of which the concept of ‘leadership’ is understood, like linking it to a ward administrative and managerial role rather than to nurses clinical roles, may stem from nursing education’s failure to convey that leadership is an integral part of the nursing role, as well as from vague clinical job descriptions linked to leadership for nurses in the clinical field. When participants in this study stated that the clinical portfolio of their curriculum offered them few opportunities to learn leadership, this may reflect inconsistencies in their perceptions of leadership and might lead them to expect learning in ward management. The present study does not reveal their expectations; however, their statements that the opportunities to learn leadership are few in clinical placement may also stem from systemic challenges in the nursing environment that affect leadership learning. It is well known that nurses may have insufficient opportunities to develop and apply leadership skills in their practice. A study drawing on an understanding of leadership as a clinical concern contributing to quality care, explored the key factors influencing the development of leadership among healthcare professionals in nursing homes [47]. The study identified several interrelated challenges within the nursing home context that hindered staff from fully realizing their leadership potential. These included insufficient time, staff shortages, and frequent turnover, which disrupt team stability; rigid management structures; negative team dynamics; inadequate onboarding; and poor communication, all of which contribute to weakened professional identity. These factors not only hinder professional growth but also highlight challenges related to learning transfer in leadership within current models of clinical supervision for nursing students. A study assessing the impact of an intervention aimed at improving the efficiency of nurse preceptor supervision of nursing students [48] noted that, because clinical environments are often dynamic and unpredictable, nurse preceptors may prioritize ensuring students’ basic competencies. As a result, nurse preceptors may underprioritize students’ leadership learning. Likewise, a study exploring how staff nurses perceive their contributions to the education of nursing students [49] found that supervising nursing students in leadership roles often required stepping back from patient care, as preceptors were overwhelmed by heavy workloads. While this does not imply a lack of leadership in their practice, it highlights that the conditions for modeling and teaching it to students can be lacking or challenging. Hence, mentorship programs, such as the one explored in this study, may serve as a valuable complement to traditional clinical supervision of nursing students during their prerequisite leadership coursework. In our study, exposure to a new and different role and function within the practice environment appears to have facilitated the third-year students’ learning or, more directly, have connected them to the concept of leadership. A concept analysis [50] identifies the defining attributes of leadership among nursing students as skills in interpersonal communication, the ability to respond knowledgeably to clinical issues, and the competence to role model for others. As shown in our findings, the mentorship experience enhanced all of these attributes among the study’s participants. Mentoring first-year students enabled them to take on a leading role explicitly. This experience not only strengthened their nursing identity and autonomy but also, as we see it, expanded their understanding of leadership beyond ‘managing the ward’.

A notable theme in our findings was the participants’ perception of their role as mentors extending beyond teaching mentees psychomotor skills. For instance, role modeling ethical conduct toward nursing home residents was a central concern for them, one they took considerable pride in. This focus on ethical behavior suggests that they were modeling a holistic approach to nursing practice. A study describing nursing students’ experiences of leadership during their final year of education [51] found that as students adopted more holistic perspectives on nursing care, leadership became more embedded in their everyday practice. The participants in this study showed a similar pattern: their sense of responsibility to deliver high-quality nursing care and advocate for nursing home residents seems to have fostered a growing sense of leadership.

In this study, the interpersonal aspects of leadership posed the greatest challenges for the participants. For instance, encountering disengaged, and, at times, rude behavior from mentees tested the third-year students’ resilience. This aligns with findings in other studies. Jacobsen et al. [26] found that mentees’ lack of interest in clinical situations poses a challenge for student mentors. A study exploring the benefits and challenges of peer student mentorship [52] found that students often struggled to balance boundaries and approachability in their mentoring of peers. As role models, they were expected to exert some authority while maintaining a friendly and supportive relationship with mentees. This dual expectation was also manifest in our study, as participants described the emotional toll of navigating relational dynamics. While some third-year students reported coping by supporting one another, their experiences underscore the complexity of adapting to leadership roles within clinical education.

Relational difficulties are not unique to peer mentorship but are a recurring aspect of professional nursing practice. According to Cooper et al. [53], interpersonal interactions with patients, families, and colleagues are common stressors in nursing. In their concept analysis of resilience in nursing, the authors argue that adapting to interpersonal stressors is a critical component of the nursing profession; therefore, it is also a skill that requires education and training. An integrative review evaluating strategies to promote resilience in nursing students [54] emphasizes the importance of reframing interpersonal challenges as learning opportunities. Thomas and Revell [54] argue that nursing education should provide structured opportunities for students to reflect on adversity, persevere in the face of conflict, and engage in simulations of difficult situations followed by guided debriefing. The relational challenges faced by participants in our study may serve as a testing ground for building resilience. However, our findings suggest that such challenges can sometimes exceed the capacity of student mentors to adapt effectively. This highlights the need for faculty-led guidance to help student mentors navigate interpersonal dynamics, not only to improve their mentorship experience but also to prepare them for the relational complexities of leadership in nursing practice.

### Implications for nursing education

The findings of this study suggest that integrating peer mentorship into the nursing curriculum offers significant opportunities to foster leadership, collaboration, and professional identity development among third-year students. Mentorship programs embedded in the curriculum, such as the one explored in this study, provide students with a unique combination of independence and structured reflection, encouraging self-discovery and confidence-building.

The group mentoring structure was particularly effective, allowing students to collaborate, reflect on care practices, and balance self-doubt with growing self-assurance. Mandatory peer mentorship in clinical placement may help foster supportive learning environments in which students can develop clinical skills, communication abilities, and nursing and caring practices. However, to maximize the benefits of peer mentorship, careful consideration must be given to how such programs are presented, structured, and supported. For example, the participants in this study emphasized the need for sufficient time to acclimatize to the clinical environment and to become better acquainted with one another before assuming mentorship roles. Scheduling mentorship activities later in the placement period could help students feel more prepared. Additionally, extending the mentorship period beyond one week may provide students with more opportunities to practice leadership and refine their skills. The findings also underscore the importance of addressing perceptions of leadership in nursing. Leadership is often viewed as an administrative rather than a relational role, which may reflect gaps in current nursing education.

The interpersonal challenges faced by student mentors highlight the need for more faculty-led guidance and structured support. Navigating interpersonal stressors is a critical skill for the nursing profession, but it requires intentional education and training. Nursing programs should provide opportunities for students to reflect on adversity, persevere in the face of conflict, and engage in simulations of difficult situations followed by guided feedback. These measures can help prepare students not only for mentorship roles but also for the complexities of professional nursing practice.

### Strengths and limitations of the study

One strength of this study is the potential relevance of its findings and insights for both national and international nursing education programs. While our goal is not to generalize but to present an interpretation of the impact of participants’ experiences as mentors in the context of clinical placements in nursing homes, we believe that the findings offer transferable value to other settings, both within clinical nursing education and other contexts.

One limitation of this study is that the transcripts may not fully capture the experiences of all participants. In focus groups, some participants may dominate the discussion while others remain quiet, which can lead to an imbalance in the data collected [30]. In this study, no participant was observed to dominate the discussions or impede others from sharing their opinions. However, in some focus groups, some participants spoke less, raising the possibility that their experiences may be underrepresented in the data. That said, it is not our impression that their perspectives are not included. The moderators actively encouraged quieter participants to share their opinions, resulting in some recordings of their views. Additionally, co-moderators observed nonverbal cues and noted whether participants appeared to agree or disagree with others’ opinions. Overall, we believe that the viewpoints of more reserved participants were adequately included in the data. For practical reasons, it was not possible to gather the sample of participants to member-check the credibility of the data in this study. Participants reviewing transcripts, findings, and reports to ensure they accurately reflect their experiences and perspectives, generally enhances the trustworthiness of a study [39].

Regarding sample size, the study included 60 participants of 331 third-year students who mentored their junior peers in nursing homes. This means that 217 students in the student cohort did not share their experiences with peer mentoring. These may have experiences or perspectives that are not captured in this study. As a result, our findings may not fully represent the entire student cohort. However, while the sample size was limited to 60 participants across nine focus groups, our analysis indicated that saturation was achieved before the final focus groups. The richness of the participants’ descriptions is a notable strength, enhancing the depth of information about their experiences and perceptions.

Another potential limitation stems from our interpretations of the data, which may have been influenced by our preconceptions and professional backgrounds. All authors are nurse educators with experience in students’ clinical placements in nursing homes and have familiarity with the curriculum. To minimize potential bias, we have rigorously adhered to the data as it emerged from the transcripts. Analyzing qualitative data requires time, skill, and careful attention to what the data reveals [33]. In this study, extensive transcripts allowed multiple approaches to analysis. Individual analyses conducted by each researcher differed slightly in focus but remained grounded in the data. Reaching consensus on the findings required multiple rounds of consideration. However, we view the research team’s diverse perspectives as a strength, contributing to a more multifaceted and nuanced understanding of the data.

Another challenge concerns the overlapping concepts and terminology in peer mentoring and educational practices. Concepts such as ‘peer learning’, ‘peer coaching’, and ‘near-peer teaching’ are often used interchangeably with peer mentorship in the literature, despite having distinct meanings in some contexts. The same applies to multiple concepts intersecting with the term mentor, such as ‘guide’, ‘coach’, ‘sponsor’, and ‘supervisor’. Throughout the literature review supporting this study and within the manuscript, we have aimed to maintain accuracy and consistency in our use of these concepts. Still, we acknowledge the possibility of inadvertent cross-use of terms.

## Conclusion

The findings suggest that this short but intensive mandatory mentorship program served as a valuable learning activity, providing third-year students with an opportunity to reflect on their knowledge, practice leadership, and build confidence in caregiving practices. By navigating the dual role of learner and mentor, participants refined their understanding of professional nursing care responsibilities and developed skills critical to their transition to the workforce.

The mentorship program stood out as a meaningful and impactful component of the third-year students’ eight-week clinical placement. Despite its brevity, the learning activity provided opportunities for independence, collaboration, and self-discovery that are frugally afforded in traditional clinical placements. The group mentoring structure, particularly, fostered peer support and reflective learning, allowing students to balance self-doubt with growing self-assurance. These findings highlight the potential of peer mentorship as a valid and effective strategy for nursing education. When thoughtfully designed and supported, mentorship activities can assist third-year nursing students in developing the skills, confidence, and professional identity needed for their transition into the workforce. Further research is required to explore mentorship activities to maximize their impact on student learning and readiness for professional practice.

## Supplementary Information

Supplementary Material 1.

## Data Availability

The datasets generated and analyzed during the current study are not publicly available due to participant confidentiality agreements but are available from the corresponding author on reasonable request.
